# Micellisation Mechanism and Behaviour of Soluplus^^^®^^^–Furosemide Micelles: Preformulation Studies of an Oral Nanocarrier-Based System

**DOI:** 10.3390/ph12010015

**Published:** 2019-01-19

**Authors:** Julia F. Alopaeus, Ellen Hagesæther, Ingunn Tho

**Affiliations:** 1 Department of Pharmacy, University of Oslo, 0316 Oslo, Norway; j.f.alopaeus@farmasi.uio.no; 2 Faculty of Health Sciences, OsloMet—Oslo Metropolitan University, N-0130 Oslo, Norway; elha@oslomet.no

**Keywords:** solubilisation, self-assembling, graft copolymer, furosemide, oral administration, nano drug carrier

## Abstract

In this study, self-assembling Soluplus^^®^^ micelles were examined for inherent properties. Through calorimetric analysis, the critical micelle concentration (CMC) could be determined at 25 and 37 °C, and the influence of three media (Milli-Q water, phosphate-buffered saline (PBS) with a pH of 7.4 and 0.1 M HCl) on the lower critical solution temperature (LCST) was detected. Furthermore, the solubilisation of a poorly soluble drug, furosemide, into the Soluplus^^®^^ micelles was studied. The concentration-dependent properties of the micellar system were assessed through an examination of the micellar size, polydispersity, morphology, viscosity and solubilising properties, which were all found to be affected by the concentration, but temperature, pH and the composition of the test medium were also found to have an effect. Homogeneity in the estimated micellar size and morphology was shown for monophasic micelle dispersions in lower concentrations and with a shift towards more complex structures or aggregates in higher concentrations. The micelles were further investigated in terms of drug release and biocompatibility with mucus-producing HT29-MTX cells, where no biocompatibility issues were found. In this research, the implications for oral drug delivery are discussed and valuable preformulation information is provided on the micellar properties of a Soluplus^^®^^ drug system in a liquid or semi-solid form.

## 1. Introduction

**Figure 1:**
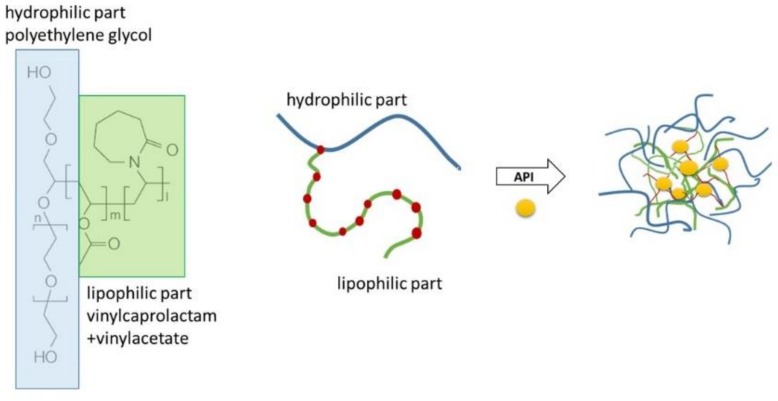
The molecular structure of Soluplus^^®^^ and a schematic representation of the formation of micelles solubilising a poorly soluble active pharmaceutical ingredient (API).

Soluplus^^®^^ (SP) is a graft copolymer consisting of polyethylene glycol (PEG), polyvinyl caprolactam, and polyvinyl acetate (13% PEG 6000/57% vinyl caprolactam/30% vinyl acetate; Figure 1) with a molecular weight ranging from 90–140,000 g/mol [1]. It has a PEG 6000 backbone with one or two sidechains consisting of vinyl acetate randomly copolymerised with vinyl caprolactam. SP was originally developed as an excipient for hot-melt extrusion [2] and has been reported in many studies to form amorphous solid dispersions [2,3,4,5]; commonly used techniques for forming amorphous solid dispersions include spray drying, electrospinning and solvent evaporation technique [5,6,7]. Due to the amphiphilic character of the polymer, it forms micelles in aqueous media (Figure 1) and can be used to solubilise poorly water-soluble drugs [8]. SP micelles have also been suggested in earlier studies as good candidates for use as nano drug carriers for intravenous cancer therapy [9], injections into arthritic joints [10] and topical delivery as semi-solid or liquid systems for transdermal or ocular delivery [11,12]. 

It is well known that the major driving force for the self-assembly of amphiphilic polymers into micelles is hydrophobic interactions that lower the free energy in the system as the hydrophobic components withdraw from the aqueous media to form a core [13,14]. This happens at a threshold level specific to different polymers, occurring above the critical micelle concentration (CMC) and critical micelle temperature (CMT). The effect of temperature in the micellisation process can be derived from the following equation, which describes the standard free energy of the micellisation process:

ΔG_mic_ = RT ln(CMC) (1)

where R is the gas constant and T is the temperature in the system. For polymers with positive entropy, the CMC will decrease with the increasing temperature. Below the CMC and CMT, in an aqueous environment, amphiphilic molecules exist separately, but when the concentration or temperature increases, unimers and micelles exist in equilibrium with an increasing tendency towards aggregation; finally, when the concentration or temperature is raised high enough, aggregation or gelation may occur [15]. There is still a need for further elucidation on the micellisation mechanism and the concentration-dependent behaviour of SP micelles in aqueous media with pH values resembling gastrointestinal conditions.

Furosemide (FM) is a Biopharmaceutics Classification System (BCS) class IV drug (i.e., poor aqueous solubility and poor permeability) with an acidic pKa value of 3.8 [16]. The aqueous solubility increases as a function of pH from 0.18 mg/mL at pH 2.3 to 13.36 mg/mL at pH 10 [16]. The drug is a loop diuretic used for the oral treatment of oedematous states associated with cardiac, renal and hepatic failure and the treatment of hypertension. FM was chosen as model drug for this study, because it is both poorly soluble and exhibits poor membrane permeability—both characteristics, which may potentially be improved with the addition of a solubiliser like SP [9,17].

The aim of this study was to provide valuable preformulation information on the micellar properties of SP micellar systems and how they are likely to respond to biologically relevant media. To our knowledge, there is incomplete understanding of SP micellar behaviour, which this study aims to address through several analysis methods. More specific aims of this study included the evaluation of the solubilisation mechanism of SP micelles in aqueous media over a range of polymer concentrations (0.5–20% *w*/*w*), different pH values (pH 1.2 and 7.4), compositions of media (0.1 M HCl, phosphate-buffered saline (PBS) and Milli-Q water) and temperatures (25 and 37 °C) using furosemide as a model drug. The objective was to further evaluate the implications for oral drug delivery with respect to these properties. In this work, we have studied the micellisation mechanisms using calorimetric analyses (isothermal titration calorimetry and solution nano differential scanning calorimetry (DSC)) and analysed the concentration-dependent behaviour of FM–SP micelles in terms of solubilising capacity, viscosity, estimation of micellar size, polydispersity and morphology, drug release and biocompatibility. The characteristics have been studied in media relevant to oral drug delivery, namely 0.1 M HCl and PBS with a pH of 7.4, in addition to purified water. Insight into its concentration-dependent behaviour is highly relevant for tailoring a liquid or semi-solid oral nano delivery system. 

## 2. Materials and Methods 

### 2.1. Materials

Soluplus^^®^^ (SP) was kindly donated by BASF (Ludwigshafen, Germany). Furosemide (FM) (Fagron, Copenhagen, Denmark) was used as a model drug. Disodium hydrogen phosphate, potassium dihydrogen phosphate and sodium chloride were obtained from Sigma-Aldrich (St. Louis, MO, USA). Methanol (MeOH) of high-performance liquid chromatography (HPLC) grade (Merck, Darmstadt, Germany) was used. The water was purified by the Milli-Q^^®^^ integrated water purification system for ultrapure water (Merck Millipore, Darmstadt, Germany) and is referred to as Milli-Q water. The theoretical pH of ultrapurified water is 6.998 [18], but as the buffer capacity of pure water is very weak, this is subject to rapid change after sourcing the water from the Millipore system, and the pH values of the Milli-Q water were therefore not reported throughout this study. All the other chemicals and solvents were of reagent grade or HPLC grade.

Additionally, 0.01 M phosphate-buffered saline (PBS) with a pH of 7.4(0.0027 M KCl and 0.137 M NaCl) was prepared from tablets acquired from Sigma-Aldrich (St. Louis, MO, USA) and Milli-Q water. For analyses where larger quantities of PBS were needed, the medium was prepared according to European Pharmacopoeia 4.1.3. [19]. In addition, the phosphate buffer with a pH of 6.8 for the mobile phase was prepared according to European Pharmacopoeia 4.1.3. [19]. 

The HT29-MTX cell line was kindly provided by Dr. Thécla Lesuffleur (INSERM UMR S 938, Paris, France). These mucus-secreting cells were adapted and cultured for several passages in a medium containing 10^−6^ M methotrexate (MTX) and reversed for several passages in a drug-free medium [20]. They do not need to be maintained in media containing MTX in order to differentiate into a mixed population of mucus-secreting goblet cells and enterocytes after confluency. The cells used in this study were from passages 24–26.

The medium for cell growth comprised the following: Dulbecco’s Modified Eagle’s Medium with high glucose (DMEM), L-glutamine, sodium pyruvate and phenol red with a pH in the range of 6.8–7.2 (sodium bicarbonate buffer), which was further supplemented with 10% inactivated foetal bovine serum, penicillin (100 units/mL) and streptomycin (100 μg/mL) from Sigma-Aldrich (St. Louis, MO, USA). The medium for the cell experiments, Hanks’ Balanced Salt Solution (HBSS), trypsin-EDTA, 5(6)-carboxyfluorescein, Thiazolyl blue tetrazolium bromide (MTT) and Triton-X were all provided by Sigma-Aldrich (St. Louis, MO, USA).

### 2.2. Preparation of Micellar Dispersions

SP was dispersed in an aqueous solution (Milli-Q water, PBS with a pH of 7.4 and 0.1 M HCl) at the desired concentration (0–20% *w*/*w*) and stirred overnight until a homogeneous appearance was obtained. FM was added to up to concentrations of 1 mg/mL or 3 mg/mL or in excess for saturated micelles. The containers were protected from light due to the photosensitivity of FM, and the dispersions were stirred until the active pharmaceutical ingredient (API) was completely dissolved or, in the case of API, added in excess for 96 h and filtered to 0.45 µm. 

### 2.3. Isothermal Titration Calorimetry (ITC) 

A Microcal PEAQ-ITC System (Malvern Instrument Ltd., Malvern, UK) was used to determine the CMC and ΔH for SP in water at 25 and 37 °C. The calorimetric titration was performed as described by Tanida [8]. The sample cell (300 µL) was filled with water, and aliquots of 1 µL SP (7.5% *w*/*w*) were injected into the sample cell. The time interval between two consecutive injections was 120 s, and the agitation speed was 300 rpm for all the experiments. Each titration was performed three times to ensure the reproducibility of the results. The data analysis was carried out using the Microcal Analysis software supplied by the manufacturer. The CMC and change in enthalpy of micellisation (ΔH_mic)_ were determined from the plot of integrated heat data according to Bouchemal [21,22]. This method determines the enthalpy of demicellisation, but because ΔH_mic_ = −ΔH_demic_, the value of ΔH_mic_ can be directly derived [21]. The Gibbs free energy (ΔG_mic_) and the entropy of micellisation (ΔS_mic_) were calculated from ΔH_mic_ and CMC with the following equations:

(2) ΔGmic = RT ln(CMC)

(3) $$\Delta Smic\;=\;\frac{\Delta Hmic\;-\;\Delta Gmic}{T}$$

Further calorimetric analyses were conducted by replacing the water in the sample cell with FM in an aqueous solution (0.065 mM), which had a molar concentration 10 times lower than that of the injected SP concentrations (7.5% *w*/*w* corresponding to 0.65 mM). A total of 39 injections of 1 µL of SP with the same time interval and agitation speed as above were performed. The titration experiment was performed with three replicates and at 25 °C. The CMC, enthalpy, Gibbs free energy and entropy of micellisation were extracted and calculated in the same way as for pure the SP micelles in Milli-Q water. 

### 2.4. Nano DSC

The change in heat capacity (ΔCp) associated with the sol–gel transition of the polymeric micelle solutions was studied using the Nano DSC Model 602000 (TA Instruments, Lindon, Utah, USA) for solutions. Samples of 5% (*w*/*w*) SP were analysed with and without 0.05 mg/mL of FM in three media (Milli-Q water, PBS with a pH of 7.4 and 0.1 M HCl). The samples were degassed for 10 min before they were placed into the cell, where they were kept for at least 10 min at 5 °C prior to analysis. Milli-Q water was placed in the reference cell. The analyses were run under constant pressure (3 atm) with a heat rate of 2 °C/min over the temperature range of 5–90 °C. Milli-Q water was used as a blank. The samples were analysed in triplicate.

The data processing was accomplished with Nano Analyze software provided by the manufacturer. The integration was carried out using the “two state scaled” model, and a sigmoidal baseline was drawn from around 20 °C to around 70 °C to obtain the transition midpoint temperature (Tm) and enthalpy of fusion (ΔH).

### 2.5. Quantification by HPLC

The HPLC system was from Shimadzu Corporation (Kyoto, Japan) and consisted of an auto-injector SIL-9A, a Prominence LC-20AD pump, a Chromatopac C-R5A integrator and a Prominence SPD-10A UV detector. The column oven was an Igloo-CIL from SCP GmbH (Weiterstadt, Germany). The separation was performed with a reversed-phase column (Nova-Pak^^®^^, C18, 4 µm, 3.9 × 150 mm, Waters, Wexford, Ireland) equipped with a Guard Column (Nova-Pak^^®^^ C18, 4 µm, 3.9 × 20 mm, Waters, Wexford, Ireland). 

The mobile phase consisted of phosphate buffer with a pH of 6.8 (see Section 2.1): MeOH (70:30 *v*/*v*). The mobile phase was filtered through a 0.45-μm membrane filter and degassed continuously with He gas during the run. The sample volume was 10 µL, and the flow rate was 1 mL/min. The column oven was set to 30 °C, and the detection wavelength was 276 nm. The retention time for FM was approximately 8 min. The calibration curve in the range of 0.50–3.75 µg/ml (*r*^2^ ≥ 0.99) was prepared from a stock solution of 1 mg/mL in MeOH and diluted with the mobile phase.

For the quantification of samples containing SP, it was essential to make sure that the polymer was sufficiently washed off the column between injections by regularly running a washing program.

### 2.6. Solubility Studies

SP dispersions (10 mL) of concentrations of 0–17% (*w*/*w*) were prepared as described above in three different media (Milli-Q water, PBS with a pH of 7.4 and 0.1 M HCl). FM was added in excess, the containers were protected from light, and the samples were stirred for 96 h at room temperature. The samples were filtered to 0.45 µm (VWR, Radnor, PA, USA), and 100 µL was diluted to 10 mL with MeOH (except for 0.1 M HCl, which was diluted in the mobile phase (for the composition, see above)) and quantified by HPLC. Each concentration was prepared in triplicate for each solvent.

### 2.7. Viscosity

The viscosity measurements were performed using a Brookfield viscometer DV2T (Middleboro, MA, USA) equipped with a Julabo ED-5 (Seelbach, Germany) temperature regulator connected to a water bath. A stainless-steel cone and plate geometry with a CPA-52Z cone for viscosities higher than 10 mPa·s and a CPA-40Z cone for lower viscosities was applied. A sample of the SP dispersion of 0.5 mL or 1.5 mL for high and low viscosities, respectively, was measured at a constant shear rate of 400 s^−1^. 

The viscosity was determined for the SP dispersions in the concentration range from pure solvent to 17% (*w*/*w*) polymer in three different media (Milli-Q water, PBS with a pH of 7.4 and 0.1 M HCl) at 25 °C. Each measurement was performed in triplicate.

### 2.8. Micellar Size, Polydispersity and Zeta Potential

Dynamic light scattering was used in order to determine the hydrodynamic diameter (size), polydispersity and aggregation of the micellar structures, employing a Zetasizer Nano Series (Malvern Instruments Ltd., Malvern, UK). The measurements were performed at 25 °C with a 173° backscatter angle for the FM-saturated SP micelles in the concentration range of 0.5%–20% (*w*/*w*) in three different media (Milli-Q water, PBS with a pH of 7.4 and 0.1 M HCl). The samples were run in triplicate, and the resulting values were counted as an average from three subsequent runs with 10 measurements each. The polydispersity index (PDI) and size distribution were followed to identify the SP concentration where the aggregation of micelles to supramolecular structures dominated.

The zeta potential of the 5% (*w*/*w*) SP micelles (FM-saturated) in Milli-Q water was assessed by microelectrophoretic measurements using the Zetasizer Nano at 25 °C, and the samples were run in triplicate.

In order to further assess the influence of temperature on the aggregation of micelles, 5% (*w*/*w*) SP micelles (FM-saturated) in three different media (Milli-Q water, PBS with a pH of 7.4 and 0.1 M HCl) was investigated over a temperature range from 25 to 37 °C. An automatic program where the sample was equilibrated for 2 min at each temperature before measuring and increasing the temperature by 1 degree was used. Each sample was measured once at each temperature point. All the samples were run in triplicate. The polydispersity index (PDI) and size distribution were recorded to identify the temperature where the aggregation of micelles to supramolecular structures dominated (PDI > 0.3). 

### 2.9. Transmission Electron Microscopy (TEM)

The size and morphology of SP micelles in various concentrations were inspected by transmission electron microscopy (TEM) using negative staining with uranyl acetate.

Negative staining was performed by placing a carbon-coated Fromvar copper grid (treated with glow discharge for 60 s) over a 40-µL drop of a sample of SP (0.5, 5, 10 and 15% (*w*/*w*) in water) for 5 min. Afterwards, the grid was washed with Milli-Q water and placed over a 20-µL drop of uranyl acetate (2% (*w*/*w*) in water) for 2 min. The excess of the uranyl acetate was blotted away with filter paper. After drying, the sample was examined using a JEOL 1400Plus Electron Microscope (JEOL Ltd, Tokyo, Japan) equipped with a Ruby camera at 120 kV.

### 2.10. In vitro Drug Release

In order to distinguish between the drug solubilised in the micelles and the free drug, thereby being able to capture the release of the drug from the micelles or supramolecular aggregates, drug release studies were conducted by placing 2.00 mL of SP micelles containing FM inside a dialysis bag (MWCO 12–14 kDa). The dialysis bag (SpectraPor Dialysis membrane, Rancho Dominguez, USA) was placed in the beaker of a USP paddle apparatus containing 500 mL of test medium (Milli-Q water, PBS or HCl). The depth of the paddle was adjusted so that the agitation did not interfere with the dialysis bag but still provided the proper mixing of the release medium at 50 rpm. The samples were tested in triplicate at 37 °C. 

The 5% (*w*/*w*) SP dispersion containing three different amounts of FM (1 mg/mL, 3 mg/mL and FM-saturated micelles after 96 h with an excess of the drug) were prepared with and evaluated in different media: Milli-Q water, PBS with a pH of 7.4 and 0.1 M HCl. The FM content for the saturated micelles in PBS was approximately 25.5 ± 0.5 mg/mL. The saturated micelles were not tested in Milli-Q water or 0.1 M HCl due to the non-sink condition for the high FM concentration in these media. The samples of 1 mL were withdrawn from the medium after 15, 30, 45, 60, 90, 120, 240 and 360 min. The samples were filtered to 0.45 µm and diluted (1:1, 1:5 or 1:10, depending on the concentration) with MeOH (except for the samples in 0.1 M HCl, which were diluted in the mobile phase) and quantified by HPLC.

### 2.11. Biocompatibility with Mucus-Producing HT29-MTX Cells

The HT29-MTX cells were incubated at 37 °C under an atmosphere of 5% CO_2_. For the preservation, the cells were passaged before reaching 80% of confluency with a solution of trypsin-EDTA. The medium was changed every 2 days. The biocompatibility studies were performed by 2 different cell toxicity assays: firstly, by using the water-soluble 5(6)-carboxyfluorescein (CF) as a paracellular marker in a transcellular permeability study and, secondly, by a cell viability assessment using 3-(4,5-dimethylthiazol-2-yl)-2,5-diphenyltetrazolium bromide reduction assay (MTT). For the purpose of the CF studies, the mucus-producing HT29-MTX cells were cultured on Transwell^^®^^ inserts (Costar filter inserts, Corning, New York, NY, USA) with a pore size of 0.4 µm and a cell growth area of 1.12 cm^2^ in a 12-well plate for 21 days at 37 °C under a 5% CO_2_ atmosphere and at an initial seeding density of 2.4 × 10^4^ cells/cm^2^. 

The Transwell setups were incubated for 24 h with 0.5 mL of the sample in the donor compartment, and 1.5 mL of medium for cell growth in the acceptor compartment. After exposure to the formulations in the donor compartment for 24 h, the cells were washed and incubated under stirring (60 rpm, 37 °C) with 0.5 mL of 15 µM CF in HBSS in the donor compartment and 1.5 mL HBSS in the acceptor compartment for 2 h, before determining the amount of permeated CF in the acceptor compartment. The amount of permeated CF was measured in triplicate using the fluorescence plate reader Victor^3^™ from Perkin Elmer (Waltham, MA, USA) at λ_ex_ 485 and λ_em_ 535 for 0.1 s on black optical bottom polystyrene Nunclon™ 96-well cell culture dishes (Nunc A/S, Roskilde, Denmark) holding 200 µL of solution. The average of the three measurements was used. 

The formulations selected for the experiment were SP micelles dispersed in cell media in concentrations of 10% and 15% (*w*/*w*) and 1 cm^2^ of a solid dispersion SP (prepared as described below) with solubilised FM 0.05% (*w*/*w*), which was gently placed on top of the cell monolayer and 0.5 mL of DMEM added on top. The SP micelle dispersions were prepared in higher concentrations and diluted with DMEM to their final concentrations to eliminate the lack of growth media being a cell stressor during the 24-h incubation. Pure DMEM was used as a negative control, and Triton-X (10% *v*/*v*) was used as positive control.

For the MTT assay, the cells were seeded on transparent 96-well plates (Costar, Corning, New York, NY, USA) at a density of 2 × 10^4^ cells/well. The cells were allowed to adhere for 24 h, and after checking the morphology with an optical microscope, 200 µL of the samples were added per well to the cell and the plate was further incubated for 2 or 24 h. The negative control was cells with fresh media, and Triton-X (1%) was used as positive control. MTT assay is the assessment of cell viability via mitochondrial viability. If the cells are viable, succinic dehydrogenase is able to transform the tetrazolium salt into formazan crystals [23]. After the end of the treatment period, the samples were replaced with 200 µL of MTT reagent (0.5 mg/mL), and the plates were further incubated for 4 h. After incubation, the reagent was removed and replaced with 200 µL of DMSO, and the plates were shaken and protected from light at 120 rpm for 15 min until all the crystals dissolved. Finally, the absorbance was measured at 570 nm and 630 nm on a Spectramax 190 microplate reader (Molecular Devices LLC, Sunnyvale, California, USA) using Softmax^^®^^ Pro data acquisition and analysis software version 7.0.2. The absorbance values for all the readings at 630 nm were subtracted from the absorbance values read at 570 nm. The cell viability, expressed as a percentage, was calculated according to the following equation:

(4) $$Cell\;viability\;\%=\;\frac{Experimental\;value-negative\;control\;}{positive\;control-negative\;control}\;\times \;100$$

Each sample was prepared in triplicate and each parallel was tested in 6 independent wells for the same experiment. 

### 2.12. Preparation of Solid Dispersion 

Additionally, 25% (*w*/*w*) SP micelles were prepared, as previously described, in Milli-Q water with 0.1% FM, and 3.5% glycerol (*w*/*w*) was added with the water content adjusted accordingly. The solution was casted in a petri dish with a wet thickness of 1000 µm. The solution was left to air dry for 24 h until the solvent completely evaporated. The prepared solid dispersion was a homogeneous film that could be cut into smaller pieces and used for analysis.

### 2.13. Statistical Analysis

All the values are shown as mean ± standard deviations. All the analyses were performed using the software program GraphPad Prism 7^^®^^ (Graphpad Software San Diego, CA, USA) with the statistical significance set to *p* ≤ 0.05.

The correlation of variables was evaluated by multivariate data analysis in a principal component analysis (PCA) using the Unscrambler^^®^^ version 9.8 (Camo ASA, Trondheim, Norway). A more detailed description of the projection methods is given elsewhere, e.g., by Esbensen [24].

## 3. Results

### 3.1. Isothermal Titration Calorimetry (ITC)

**Figure 2:**
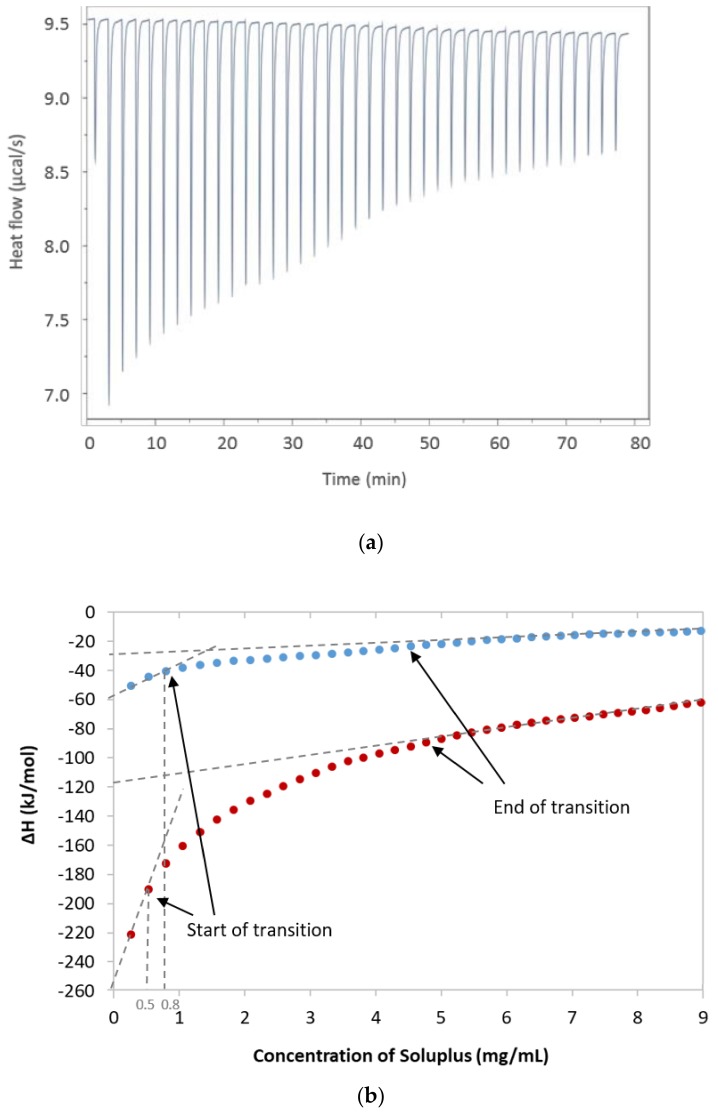
Data obtained from the isothermal titration calorimetry (ITC) analysis. (**a**) Exothermic heat flow upon injections of Soluplus^^®^^ into water at 25 °C, (**b**) Integrated heat data with indicated start and end of transition (i.e., micellisation of Soluplus^^®^^ at 25 °C); the blue dots are only Soluplus^^®^^ and the red dots are with furosemide added to the system. The dotted lines indicate on the x-axis the critical micelle concentration (CMC; concentration at the start of transition); the intercept with the y-axis was used to estimate ΔH_mic_ (ΔH_end of transition_ − ΔH_start of transition_).

Characteristic exothermic heat flow curves were obtained upon injecting small amounts of SP micellar dispersion into water (Figure 2a). After each new injection of SP, the heat flow effect was reduced as a result of the reduced concentration difference between the syringe and the cell [21]. The integrated heat data in Figure 2b displays the heat as a function of the SP concentration at 25 °C for micellar dispersion when the receiving cell contained pure Milli-Q water or an aqueous solution of FM. The thermograms were analysed as described by Bouchemal [8,21], which allowed for a rough estimation of the start and end of the transition reflecting the micellisation of SP. The start of micellisation or the critical micelle concentration (CMC) was estimated to be approximately 0.8 mg/mL at 25 °C and 0.5 mg/mL at both 37 °C and 25 °C when FM was added to the system. 

The linear fit to the thermogram (Figure 2b) was used to determine the enthalpy of the micellisation, ΔH, as the difference between the two intercepts with the y-axis. The values of the CMC and change in enthalpy were further used to calculate the Gibbs free energy of micellisation and entropy. The results are summarised in Table 1. The calculated Gibbs free energy was found to be negative and similar for all three systems tested. The Gibbs free energy is directly correlated to the CMC, and both decreased with the increasing temperature and the addition of FM.

### 3.2. Nano DSC

**Figure 3:**
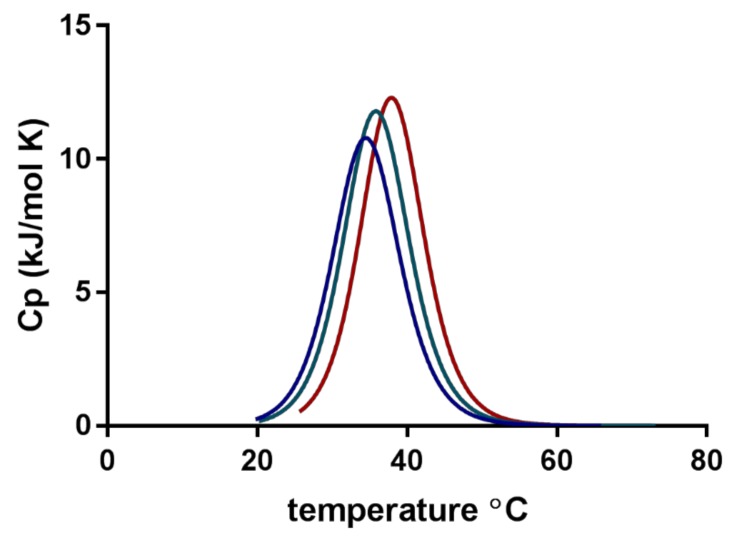
Normalised specific heat capacity (ΔCp) obtained from nano differential scanning calorimetry (DSC) of furosemide-loaded Soluplus^^®^^ micelles in three media: phosphate-buffered saline (PBS) with a pH of 7.4 (blue), Milli-Q water (green) and 0.1 M HCl (red).

Figure 3 shows the normalised changes in heat capacity (ΔCp) describing the association of individual micelles in their transition to form a cluster of micellar networks into continuous gelation for three media. The transition midpoint temperature (Tm) or the lower critical solution temperature (LCST) was shifted from 32.8 ± 0.6 °C in PBS with a pH of 7.4 to approximately 3 °C higher temperatures for Milli-Q water and 5 °C higher for 0.1 M HCl (Table 2). Experiments with pure water (no FM) showed a slight shift toward higher temperatures as compared with the micelles with FM. The ΔH was found to be between 100 and 250 kJ/mol for all the experiments, but the observed variation between the parallels made it difficult to extract specific correlations or trends. It can be mentioned that experiments with lower SP concentrations (yet above CMC) generally resulted in reduced ΔH (data not shown).

### 3.3. Solubility of Furosemide

**Figure 4:**
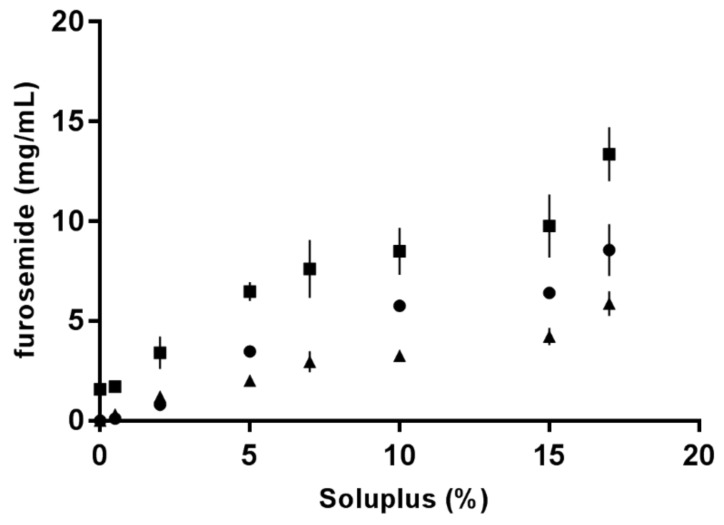
Increase in furosemide solubility as a function of Soluplus^^®^^ concentration in various aqueous media: Milli-Q water (dots), PBS with a pH of 7.4 (squares) and 0.1 M HCl (triangles).

FM has pH-dependent and limited solubility in aqueous media [16]. An increase in FM solubility as a function of SP concentration was determined using a phase solubility setup first described by Higuchi and Connors [25]. The solubilisation capacity of SP micelles in different aqueous media is shown in Figure 4, where it can be observed that FM was least soluble in 0.1 M HCl (pH 1.2) and most soluble in PBS (pH 7.4). SP was demonstrated to be an efficient solubiliser for the poorly soluble drug, FM, in all tested media. The highest solubility was achieved for 17% SP (*w*/*w*) in PBS.

### 3.4. Viscosity 

The viscosity of the FM-saturated SP micelles showed only a moderate increase up to a concentration of 7% (*w*/*w*), after which the viscosity increased more rapidly and the differences in the three tested media became more apparent. HCl solutions showed the highest viscosity at high SP concentrations followed by PBS and, finally, Milli-Q water with the smallest viscosity increase. 

### 3.5. Micellar Size, Polydispersity and Zeta Potential

**Figure 5:**
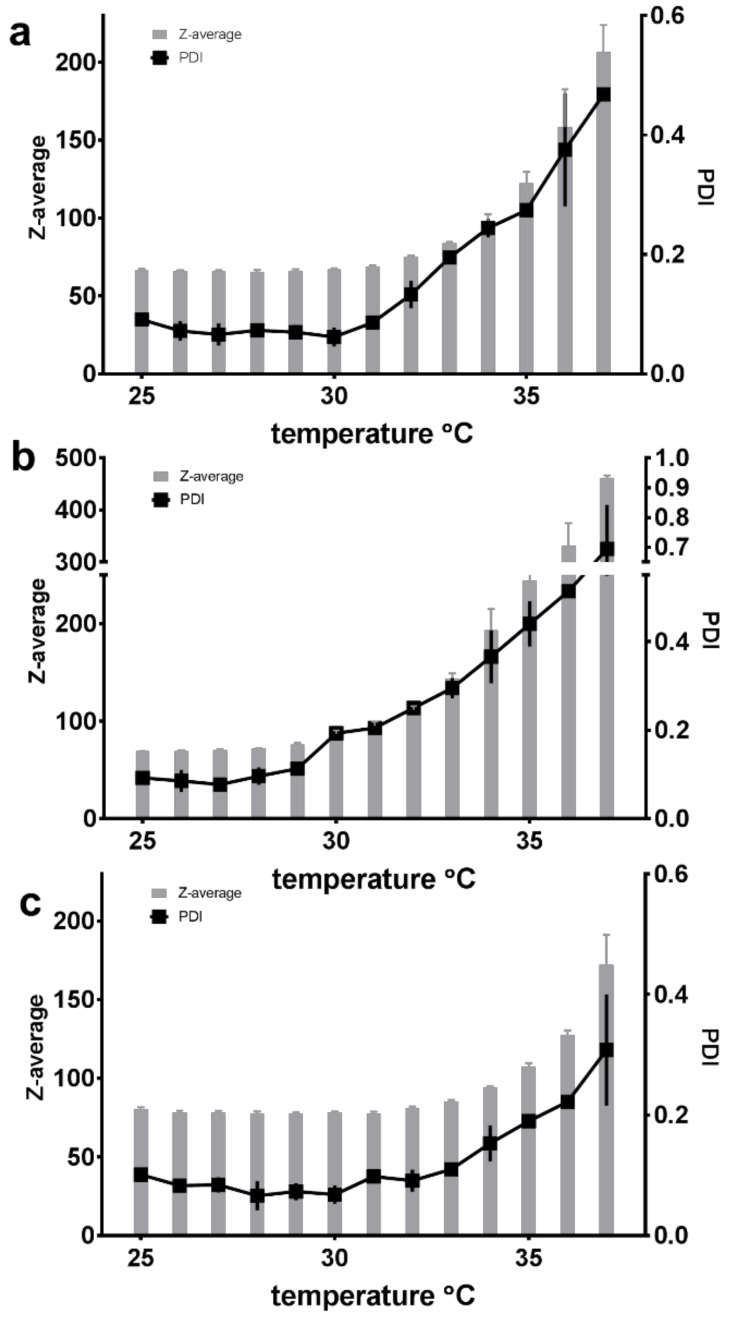
The measured Z-average and polydispersity index (PDI) of 5% (*w*/*w*) Soluplus^^®^^ micelles saturated with furosemide at temperatures of 25–37 °C in three different media: (**a**) Milli-Q water, (**b**) PBS with a pH of 7.4 and (**c**) 0.1 M HCl (*n* = 3).

Dynamic light scattering (DLS) was used to estimate the size of the micelles and the polydispersity of the system. The Z-average, interpreted as the hydrodynamic diameter of the micelles, and the polydispersity index (PDI) of the SP dispersions in all the tested media (Milli-Q water, PBS and HCl) showed quite constant values in temperatures ranging from 25 °C to approximately 30 °C for a formulation of 5% (*w*/*w*) SP micelles saturated with FM (Figure 5). With a Z-average of approximately 60–80 nm and a PDI between 0.1 and 0.2, this indicated a homogeneous population of micelles with a monophasic size distribution. An increase in polydispersity (to PDI values above 0.3) occurred at around 33 °C for the micelles in PBS, around 36 °C for those in Milli-Q water and around 38 °C for those 0.1 M HCl. The largest temperature-promoted change was observed for the SP micelles in PBS (pH 7.4), where the increase from 25 to 37 °C resulted in a size change from 70 nm to 450 nm and a PDI increase from 0.1 to 0.7 (Figure 5b). 

**Figure 6:**
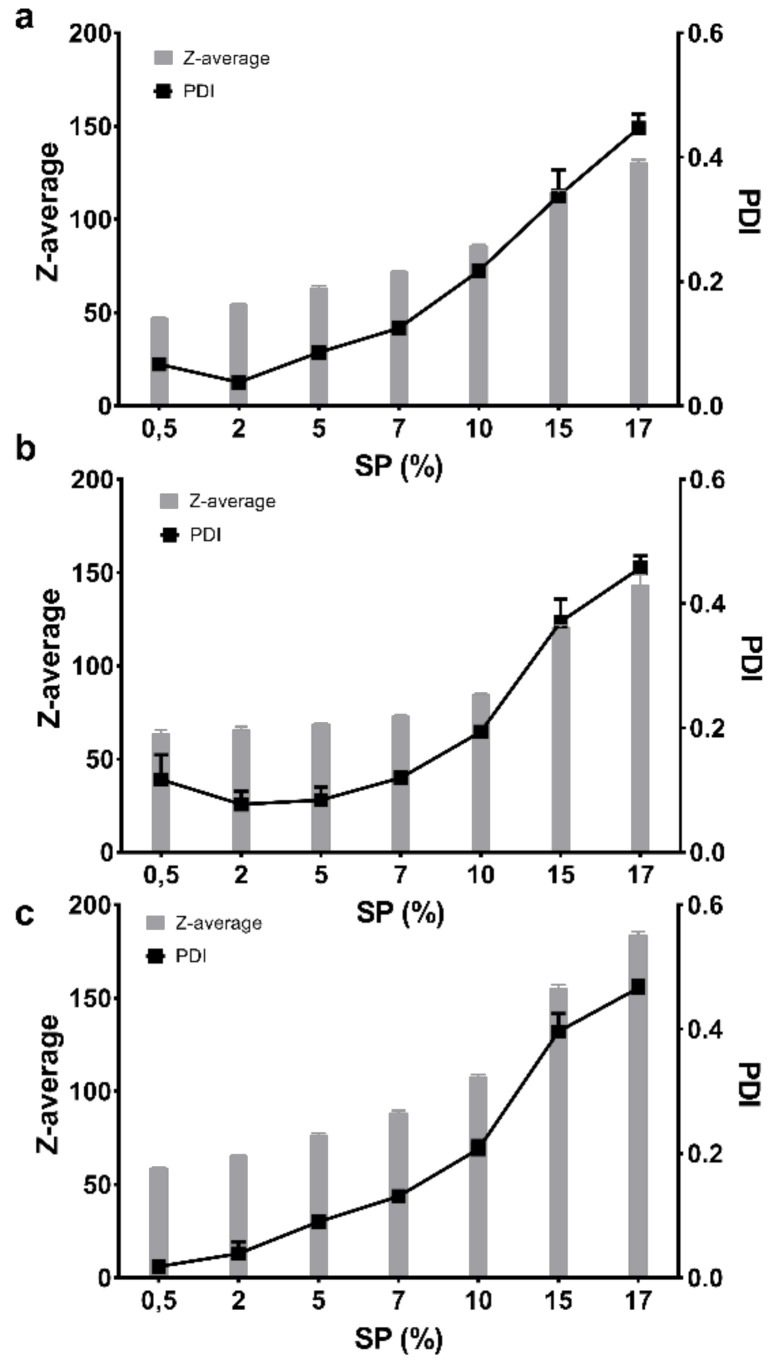
The measured Z-average and polydispersity index (PDI) at rising concentrations (0.5–17%) of the furosemide-saturated Soluplus^^®^^ in three different media at 25 °C: (**a**) Milli-Q water, (**b**) PBS with a pH of 7.4 and (**c**) 0.1 M HCl (*n* = 3).

The size and polydispersity of the FM-saturated micelles were also studied as a function of SP concentration at a constant temperature of 25 °C, which also allowed for the influence of the three media to be compared. It was observed that up to approximately 7–10% SP (*w*/*w*), the Z-average only slightly increased within one medium and the PDI remained between 0.1 and 0.2, after which the micelles in all three media experienced a rapid increase in both size and polydispersity correlated to the increasing concentration (Figure 6).

The zeta potential of 5% (*w*/*w*) SP micelles was measured to be −1.74 mV ± 0.24mV in Milli-Q water at 25 °C.

### 3.6. Transmission Electron Microscopy (TEM)

**Figure 7:**
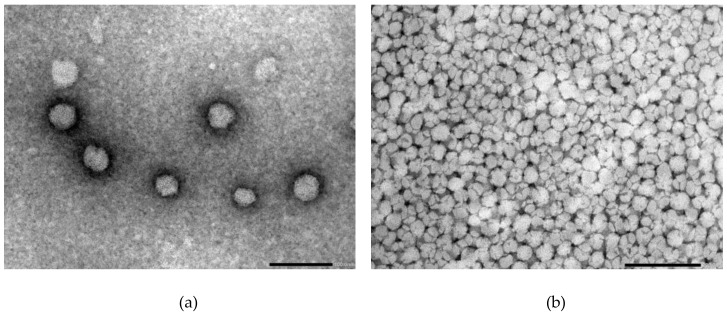

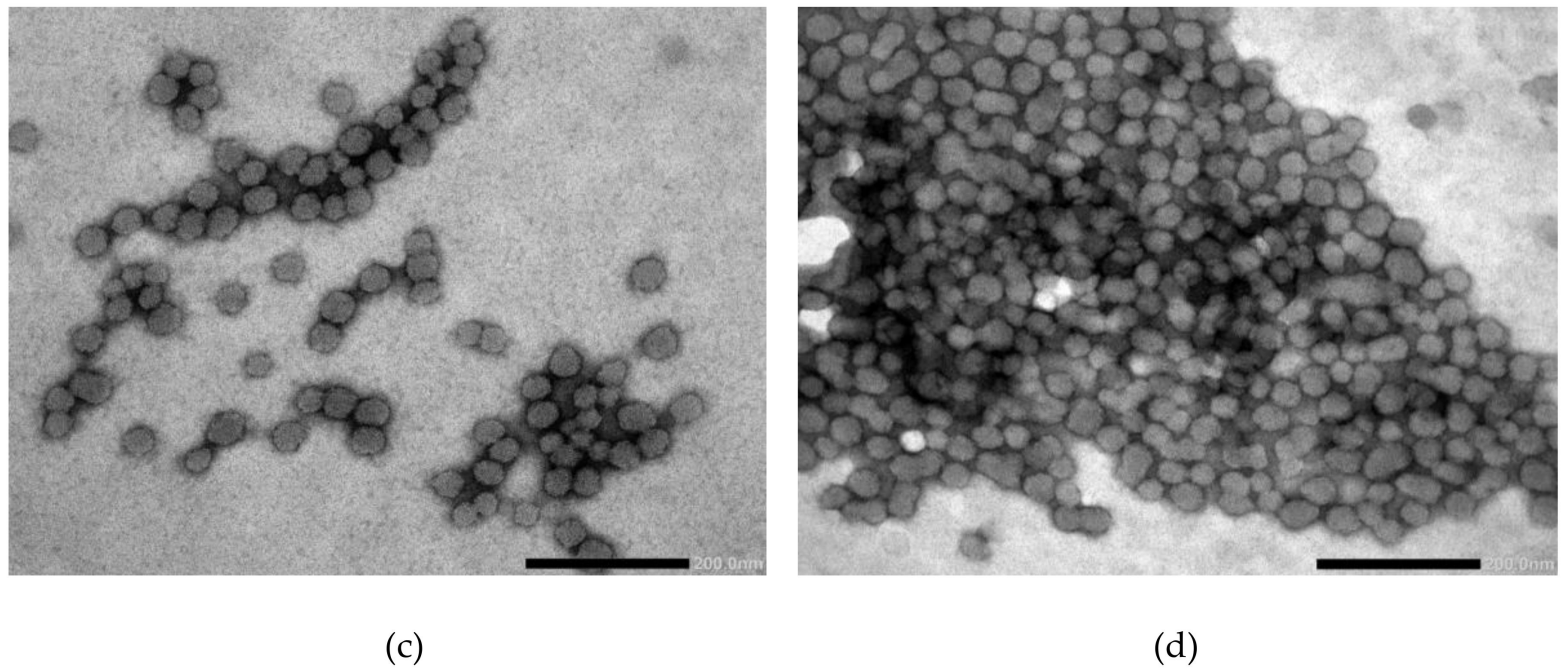
TEM images of the Soluplus^^®^^ micelles dispersed in different media at different concentrations without API (**a**–**b**) and with FM (**c**–**d**): (**a**) 0.5% SP in Milli-Q water, (**b**) 15% SP in Milli-Q water, (**c**) 1% SP in 0.1 M HCl and (**d**) 15% SP in 0.1M HCl (all *w*/*w*). The size of the bar indicates 100 nm in (**a**) and 200 nm in (**b**)–(**d**).

The morphology of the SP micelles was examined using TEM with negative staining, where a spherical shape and homogeneous appearance were confirmed in all the examined SP concentrations and media with and without FM (Figure 7a–d). There was no noticeable difference in size or appearance in the micelles saturated with FM, various concentrations or disperse media. The micellar size in all the concentrations was observed to be somewhere between 50 and 100 nm. 

### 3.7. In vitro Drug Release

In order to be able to differentiate between the drug solubilised in the SP micelles and the free drug, a release study was conducted using a dialysis bag (MWCO 12–14 kDa). The release profiles of FM from the SP micelles in the various media were very different. The release in PBS (pH 7.4) showed the fastest and highest release for all the tested formulations. The release in Milli-Q water was slow, whereas the release in 0.1 M HCl (pH 1.2) was below the detection limit of the method (data not shown).

**Figure 8:**
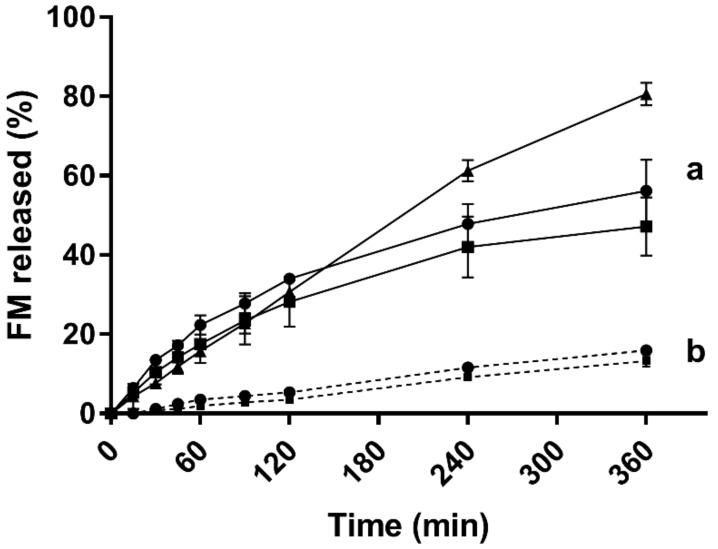
In vitro release of furosemide from the 5% (*w*/*w*) Soluplus^^®^^ micelles over the dialysis membrane (MWCO 12–14 kDa) in two different media at 37 °C: (**a**) PBS (pH 7.4) and (**b**) Milli-Q water (concentrations of furosemide: 1 mg/mL shown as squares, 3 mg/mL shown as dots and saturated micelles (25.5 ± 0.5 mg/mL) shown as triangles) (*n* = 3).

The amount of the drug loaded into the micelles was found to have a certain impact on the release rate and extent. In Milli-Q water, the release was slow, and after 6 h, between 12% and 15% of the drug load was released from the micelles loaded with 1 and 3 mg/ml of furosemide (Figure 8b). No statistical differences was observed between the two drug loads. 

In PBS (Figure 8a), a close to 80% release was obtained with the FM-saturated micelles and slightly less than 60% for the lower concentrations. For the first 2 h, the release from the three different drug loads followed the same profile, and no statistically significant differences were seen in the drug release at this stage. 

### 3.8. Biocompatibility with Mucus-Producing HT29-MTX Cells

**Figure 9:**
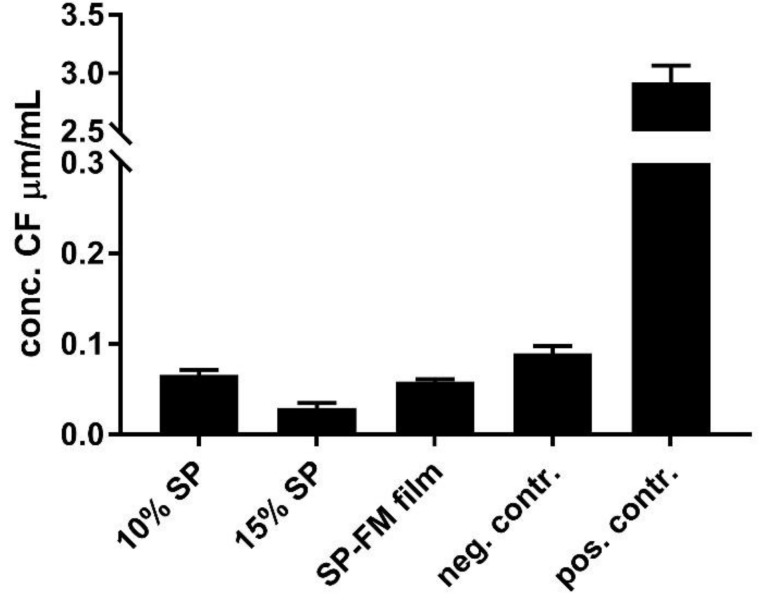
Biocompatibility assessment of various Soluplus^^®^^ micelles towards mucus-producing HT29-MTX cells shown as permeability of the water-soluble paracellular marker 5(6)-carboxyfluorescein after exposure to the formulations for 24 h. SP: Soluplus^^®^^ micelles; SP–FM film: solid dispersion of Soluplus^^®^^ and furosemide; negative control: HBSS; positive control: Triton-X.

The biocompatibility and toxicity on cells were evaluated by assessing the permeability of the water-soluble paracellular marker 5(6)-carboxyfluorescein (CF) in a Transwell^^®^^ setup using mucus-producing HT29-MTX cells, as well as through the assessment of cell mitochondrial activity using Thiazolyl blue tetrazolium bromide (MTT) to indicate cell viability. The permeability of CF after exposure to the different SP samples is presented in Figure 9. The permeability of CF is slightly reduced after exposure in all the samples compared with the negative control of the unexposed cells.

**Figure 10:**
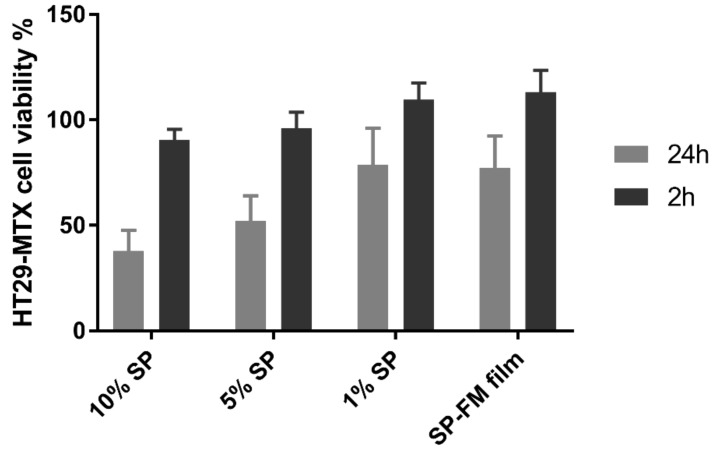
Cell viability as a cytotoxicity assessment of different concentrations of Soluplus^^®^^ micelles after exposure for 2 and 24 h in the 3-(4,5-dimethylthiazol-2-yl)-2,5-diphenyltetrazolium bromide reduction (MTT) assay. SP: Soluplus^^®^^ micelles; SP–FM film: solid dispersion of Soluplus^^®^^ and furosemide.

The cell viability results are shown in Figure 10, with cells expressing viability percentages ranging between 40–80% after 24 h of exposure. A higher SP concentration resulted in a lower viability. After 2 h of exposure, the viability in all the samples was close to 100%, indicating high viability at short exposure times.

### 3.9. Principal Component Analysis (PCA)

**Figure 11:**
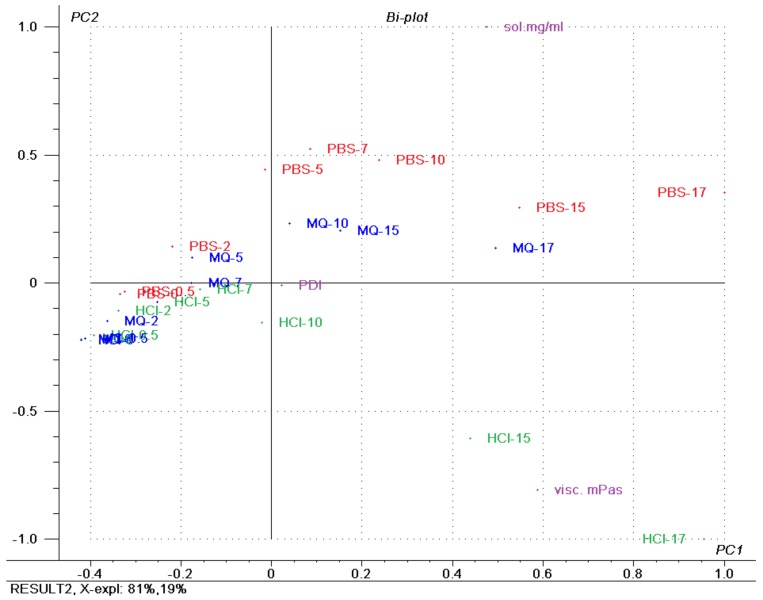
Bi-plot showing the scores and loadings superimposed, with coloured groupings of samples according to the dispersion medium, of a principal component analysis of Soluplus^^®^^ micelles containing furosemide in three media (Milli-Q water, PBS (pH 7.4) and 0.1 M HCl). The numbers included in the sample name specify Soluplus^^®^^ concentration. PDI: polydispersity index; sol mg/mL: solubilised furosemide in mg/mL; visc mPas: viscosity in mPas.

A PCA was performed on all variables. Figure 11 shows the bi-plot of the response variables of solubility (mg/mL), polydispersity index (PDI) and viscosity (mPas·s), explaining 81% of the variation on the first principal component (PC1; i.e., x-axis) and the remaining 19% on the second principal component (PC2; i.e., y-axis). The bi-plot of the scores and loadings allows for the interpretation of the correlation between the samples and the variables. The most notable observation of the PCA was the clustering of the low-concentration samples, which indicated that the variance between these samples was low and they are likely to behave in a similar manner regardless of the solubilisation media with respect to the inspected variables. At a high concentration of SP, the samples spread out more in the bi-plot, and the impact of the media becomes more apparent, correlating to higher solubility of drug and higher viscosity.

## 4. Discussion

### 4.1. Micellisation Mechanism of Soluplus^^®^^

According to a general understanding of amphiphilic molecules, SP will form micelles in aqueous media at concentrations above the CMC and temperatures above the CMT [15]. It is expected that the hydrophobic segments, the vinyl acetate with the vinyl caprolactam, would be located in the core of the micelle, whereas the strongly hydrated PEG segment would primarily be located in the outer region of the micelles. The negative Gibbs free energy confirmed that the micellisation of SP in water is a spontaneous process above the CMT and that the process of micellisation is endothermic, both with and without FM in the sample cell. The thermograms did not have a clear sigmoidal shape, which made the start and end of the transition difficult to estimate, although it is known that the exact end of micellisation can sometimes be difficult to determine for thermosensitive surfactants [22]. Furthermore, because the exact molecular weight or distribution of the polymer is not known, only the range of 90,000–140,000 g/mol, the sample must be expected to be rather polydisperse and this can influence the thermograms, as well as the accuracy of the data. 

The CMC, determined at the start of micellisation was estimated to be approximately 0.8 mg/mL for SP at 25 °C, which is in agreement with the findings of Tanida and co-workers who reported the CMC for SP in water at 25 °C to be 0.82 mg/mL [8]. They confirmed the CMC with surface tension determination. A lower CMC (0.5 mg/mL) was estimated at 37 °C and at 25 °C with FM in the receiving cell as well. A reduction in the CMC with increasing temperature has also been observed for pluronic F127 [15], and according to the free energy of micellisation equation described earlier, this is expected for polymers where ΔH is positive [13]. Nevertheless, the CMC values identified appear to be 100 × higher than the CMC given by the manufacturer at 7.6 mg/L [1] without further specification of method, solvent or temperature. Various methods can be used for the determination of the CMC, and the solvent, as well as the temperature, can affect the value in different ways. For instance, Shi and co-workers used the pyrene probe method and reported CMC values for SP of 0.61, 1.24 and 0.91 mg/L in PBS of various pH values and a value of 11.7 mg/L for CMC in 0.1 M HCl, showing both considerably lower, but also higher, CMC values than the values reported by the manufacturer [1,2]. 

The entropy of the micellisation (TΔS_mic_), as well as ΔH, with FM in the cell was also positive and much higher than without FM, although ΔG_mic_ was in the same order of magnitude. Enthalpy–entropy change follows a linear correlation in isothermal micellar formation, so the change in one will invariably result in the change of the other, which means that the effect on the free energy might not be so considerable [26]. The negative value of ΔG and the fact that TΔS_mic_ > ΔH_mic_ mean that the micellisation process can be described as entropy driven with predominance of hydrophobic interactions [22]. It may be concluded that less of the hydrophobic surface area of SP was exposed with FM present. This seems logical, because the hydrophobic areas would be expected to be associated with the hydrophobic drug in the micelle core. The positive entropy is suggested to occur when water molecules in hydration shells around the hydrophobic parts of the unimeric amphiphiles are released as a result of the micellisation process. This agrees well with the description provided by Wu et al., noting that the sol–gel transition of SP happens via hydrophobic interaction between chain segments and hydrogen bond disruption between the water and PEGylating chains at the elevation of temperature [10]. 

**Figure 12:**
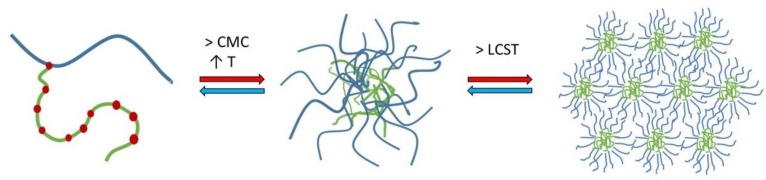
Representation of micellisation mechanism for Soluplus^^®^^ in aqueous solutions: unimers forming micelles above the critical micelle concentration and temperature and finally aggregating above the lower critical solution temperature.

Experiments with nano DSC provided insight into the changes in heat capacity (ΔCp) associated with the transition of SP micelles into aggregates forming gel networks. The transition midpoint temperature (Tm) describes the lower critical solution temperature (LCST). The LCST was found to be dependent on the composition of the media (i.e., type and content of ions including pH; Figure 3), polymer concentration and, to a lesser degree, the presence of the drug in the aqueous media. The LCST was shifted from around 33 °C for the FM–SP micelles in PBS (pH 7.4) to around 36 °C in Milli-Q water up to around 38 °C in 0.1 HCl (pH 1.2). Comparing the LCST from the nano DSC with the results from DLS as a function of temperature (Figure 5), a polydispersity index (PDI) of 0.3 was noted at approximately 33 °C in PBS, at 35–36 °C in Milli-Q water and at 37 °C in 0.1 M HCl. This suggests that the association of the polymer itself is influenced by the ionic type, content and pH of the media that affect the mechanism of micellisation (e.g., the hydration and swelling of the polymer in the medium will be affected as could be the electronic charge [10,27] or more complex supramolecular structures or gel forming networks above the LCST (Figure 12). This can also be recognised by the viscosity changes reported in Table 3. The process of micelles self-assembling from unimers forming aggregates and gels is dependent on temperature, polymer concentration and type and content of ions in the surrounding medium, and this is a reversible process (Figure 12) [13,15]. The calorimetric analyses further elucidated the thermodynamic behaviour of the SP micelle aggregation and gel formation. The CMC of SP is low, below 0.1% *w*/*w*, as identified by ITC analyses. The entropy-driven micelle formation suggested dehydration of the PEG chains to allow for the formation of entangled networks of micelles, and the results from the nano DSC revealed that LCST was dependent on the medium, ranging from around 33 °C in PBS to 36 °C in water and 38 °C in HCl. These findings are in agreement with Wu and co-workers, who suggested that the transition from solution to gel phase, also called the sol–gel transition, for SP is determined by the SP concentration, types and strengths of ions, the pH of the medium and temperature [10]. For 20% SP in PBS with a pH of 7.4 (no drug), they reported a sol–gel transition at 37 °C and slightly higher temperatures for lower SP concentrations. They further described the association of SP in three steps: the micellar formation at low concentrations takes place above the critical aggregation concentration and the critical aggregation temperature. Then, at temperatures above the critical aggregation temperatures but below LCST, they suggested that the PEG chains of the micelles dehydrate, and finally, at temperatures above LCST, the gelation of micelles occurs [10]. The entanglements of micelles into a continuous gel network at LCST are beneficial to minimise free energy [28]. Wu et al. further suggested that salts in the systems could grab the water molecules from the polymer sols and bind water tightly, making the hydrophobic segments of the polymers more likely to be excluded from the water-solvation shells and to promote the self-hydrophobic interaction [10]. This behaviour correlates well with our observations.

The enthalpy of micellisation (ΔH_mic_) was highly varying between parallels (Table 1), which may be a result of the variable molecular weight of SP. The integration was challenging due to a continuously changing baseline at higher temperatures. Nevertheless, the calorimetric enthalpy and the calculated van’t Hoff enthalpy (not shown) were mostly in good agreement for each parallel (within ±10% or less). It is not straightforward to compare the ΔH determined in ITC with those obtained by nano DCS, because the experiments are principally different. In ITC, the temperature was kept constant and the SP concentration was changed, whereas in nano DSC the SP concentration was kept constant and the temperature was changed. 

### 4.2. Concentration Dependency of Soluplus^^®^^ Micellar Behaviour 

Studies conducted at ambient temperature determined the CMC at 0.8 mg/mL, corresponding to 0.08% *w*/*w* (0.5 mg/mL at 37 °C). The solubility studies showed that the aqueous solubility of FM could be considerably increased when SP was added at concentrations above the CMC (the solubility studies were conducted at ambient temperature). Increasing amounts of FM could be solubilised with increasing concentrations of SP in all three aqueous media of different compositions and pH values. The excellent solubilising capacity of SP in water has also been reported by others [29,30]. The highest solubility isotherm was identified in PBS (pH 7.4) and the lowest in 0.1 M HCl (pH 1.2), which reflects the solubility of FM at the respective pH conditions, with one medium being well above and the other well below FM’s pKa of 3.8 (carboxylic acid group) [16]. As discussed above, the polymer will be influenced by the ionic content, types and pH of the surrounding media. At pH 1.2, SP has been shown to form salts with basic drugs, such as ketoconazole and clotrimazole, contributing to a strong increase in the solubility of the drug [27]. The carboxylic acid group of FM would be expected to be protonated at pH 1.2, but the amine group in the alpha position to the sulphur atom might also be protonated and could engage in salt formation with the negatively charged polymer. Therefore, the relative solubility increase in the acidic environment might be larger than that at neutral pH, where the drug itself is dissociated and more freely soluble. Furthermore, in PBS, the composition of salts might also contribute to the increased solubility of FM.

The shape of solubility isotherms was similar and non-linear for the three media, except maybe from 0.5% up to 5 or 7% (*w*/*w*). From 7% to 15% (*w*/*w*), a plateau with a slightly increasing slope was observed, and from 15% to 17% (*w*/*w*), the saturation concentration of FM in the micelles increased markedly in all three media. Increasing the polymer concentration generated more micelles that solubilised more FM up to above 10 to 15%. Above which, the solubility markedly increased, as did the viscosity and the polydispersity of the samples. Seen together, these findings suggested that the concentration of micelles was so high and close in proximity that the micelles started aggregating and forming clusters or larger supramolecular structures [31]. An aggregation was confirmed by the PDI values that showed a rapid increase at high polymer concentrations. It is not unlikely that such aggregates could further contribute to an increased solubility of FM, as was observed in the FM–SP micelles with concentrations above 15% SP. The aggregation or association of micelles to each other was also recognised by the increased viscosity. The exact SP concentration from which the viscosity markedly increased was different in the three media, but the tendency was similar. The highest viscosity was noticed in HCl followed by PBS. The electrolytes present in these media clearly affect the interaction points of the polymer, supporting some entanglement or aggregate formations between the micelles. The entanglement of micelles into a continuous gel network was found to be mainly an effect of temperature also influenced by the SP concentration and type of medium. Other authors have also reported on the effect of temperature and salts on the viscosity of SP [32]. Nevertheless, the results from dynamic light scattering clearly showed that at 25 °C, the micelles were monodispersed with similar size of 60–80 nm in all three media at a low concentration of SP (5%). The size of the micelles was confirmed by TEM and is in the same order of magnitude as has been reported for SP micelles by others [8,10,30]. Increasing temperature and concentration resulted in higher polydispersity in all media, and what was measured by dynamic light scattering was probably no longer single micelles, although the Z-average reported larger sizes. The TEM micrographs revealed that single micelles could be recognised also at higher concentrations, but that they were much closer located and might occur in higher numbers. Based on the fact that the association and micellisation of the polymer is affected by the ionic type and content (including pH) of the surrounding medium combined with the corresponding observed viscosity increase, it is likely that clusters of single micelles were measured by dynamic light scattering. The observed viscosity increase would be caused by an increased number of interactions between the micelles in media with higher ionic content (PBS and HCl). Hence, it is possible that single micelles are behaving as larger units [15]. It should, however, be mentioned that the sample preparation method, especially the drying, might influence the appearance of the TEM micrographs, and it was noticed that the clustered micelles appeared in samples of both high and low polymer concentration. 

Because it has been described in the literature that SP samples at concentrations below 20% do not show viscoelastic properties but behave as Newtonian fluids [28], the current study was limited to viscosity measurements. 

The PCA of solubility, viscosity and PDI emphasised that these parameters are strongly correlated in low-concentration FM-saturated SP formulations (Figure 11) and that in samples with SP concentration above 10% (*w*/*w*) the effect of dispersed medium is markedly increased with increased concentration. The type of medium has an impact when it comes to the solubilisation of a drug, where the pH of the medium relative to the pKa dictated the rank order, as well as an effect of the electrolyte salts. For PDI, no effect was found in the tested variables, and for viscosity, the type of medium had an effect, with HCl having the largest effect.

### 4.3. Implications for Oral Drug Delievry

The CMC of SP is very low; nevertheless, there are positive effects from increasing the polymer concentration in a formulation to well above the CMC with regards to the solubilisation capacity, as well as the viscosity and texture. From an application point of view, an oral drug formulation based on SP micelles should either have a concentration below 10% *w*/*w*, where the micelles behave as single units in the nano-range area and the viscosity is low, or have a high concentration, above 17%, where there is an entangled network of micelles with a semisolid or a gel-like texture, with a significantly higher solubilisation capacity as the major benefit. Our findings suggest that the solutions will turn into gels at body temperature, and both acidic conditions in the stomach and phosphate-buffered electrolytes with higher pH values closer to the small intestine will promote entanglement and aggregation. 

SP is described by the manufacturer as practically tasteless, though a taste-masking system may be used to mask the usually unpleasant taste of the API [1]. SP has previously been investigated as a taste-masking agent itself, but due to its high aqueous solubility in physiological pH, it is likely to behave relatively poorly as a taste-masker without the addition of additional excipients [33]. A combination of sweetener with an aroma might be desired to make the taste of an oral dosage more appealing.

The zeta potential was investigated through DLS and showed that the micelles have a close to neutral surface charge, albeit on the slightly negative side. The near neutral charge is not unexpected, because the hydrophilic PEG on the outer surface is commonly added to grafted copolymers for its neutral charge to lessen the effect of electrostatic interactions [34].

Because the release test was performed with the formulation kept inside a dialysis bag, where only the drug (330.75 Da) was assumed to pass through the membrane (MWCO 12–14 kDa), the furosemide measured outside the dialysis membrane could be distinguished from the furosemide solubilised in the polymeric micelles. The release was linear over 6 h for Milli-Q water (R^2^ = 0.991) for micelles loaded with 1 mg/mL and 3 mg/mL FM and for the FM-saturated micelles in PBS (R^2^ = 0.993). For micelles loaded with lower amounts of FM (1 mg/mL and 3 mg/mL, respectively), the release profile in PBS decreased with time, and a plateau was formed, even though the test was performed under sink conditions. This suggests that the affinity of FM was higher inside the SP micelles or even in the microenvironment formed inside the dialysis bag as compared with the bulk medium on the outside. The in vitro release studies indicated that very little FM (under the detection limit) would be released in the acidic environment of the stomach. This can be explained by the low solubility of the drug at a low pH, but also by the increased ionic interactions and possible salt formation between FM and SP in pH 1.2. Once swallowed, the formulation passes through the oesophagus to the stomach. A liquid or semi-solid oral SP formulation will probably pass through the sphincter pylori out of the stomach independently of gastric emptying. Depending on the volume of liquids already present in the stomach, which will be affected by the fasted or fed state, as well as the type and amount of food and drinks, the formulation may be further diluted. On the other hand, if limited liquids are present, an entangled gel network might form when exposed to a pH of 1.2 at 37 °C. The SP–FM micelles might be expected to release very little amounts of the drug under these conditions, because the solubility of the drug is much higher inside the micelles than outside. The drug release from SP–FM micelles should be accelerated when it reaches the physiological conditions of the small intestine because of the pH and the increased electrolytes. The transit time through the small intestine is estimated to be 3–6 h [35], and based on our findings, during this period, the release from the SP micelles should be controlled and close to zero order kinetics. By employing a high drug load, even with drug-saturated micelles, a relatively high extent of drug may be released (e.g., the FM-saturated micelles released around 4 mg per hour in PBS (pH 7.4)). Even considering solid dosage forms consisting of SP solid dispersions, whether they are prepared by hot-melt extrusion, spray drying or electrospinning, they will eventually disintegrate and dissolve into the gastrointestinal fluids [4,5,7,36]. The micellar SP systems that then form could be expected to behave somewhat similarly to the mostly liquid formulations described in this study and thereby be dependent on the polymer concentration and the pH, as well as the composition of the media at the site. However, exactly how the behaviour would be in physiological conditions might still need further elucidation. For instance, it is not known whether SP will form some sort of mixed micelles with lecithin and taurocholates from the bile, how the micelles will interact with proteins and enzymes of the gastrointestinaltract or how these biological components will affect the stability and release properties from the micelles.

The CF permeation experiments with the mucus-producing cells indicated that the SP formulations were non-toxic towards a mucus-coated epithelium layer. Pre-treatment with formulations actually decreased the paracellular transport of the marker compared with the negative control. SP is marketed as a permeation enhancer by the manufacturer, so the results are, as such, surprising [1]. This finding might be explained by an interaction between SP and the mucosal layer on top of the HT29-MTX cells. It is known that HT29-MTX cells start to differentiate postconfluently into a mixed population of mucus-producing goblet cells and enterocytes after approximately 7 days in culture and are ready to use after 21 days when there is a distinct mucus layer formed on top of the monolayer [37]. The mucus-layer in itself is not reported to have a negative effect on the permeability of the hydrophilic compounds [38], but mucus in combination with SP might. It is also possible that SP acts as somewhat of an irritant on the cell monolayer [39], causing the monolayer to tighten or the cells to start producing more mucus, therefore increasing the likelihood of an interaction with SP and, thus, finally decreasing the permeability of the paracellular marker. SP is reported to increase drastically in viscosity, forming a gel-like structure, partly because of the temperature and the effect of the salts present in the cell medium [32]. This viscosity-increasing effect was also demonstrated in this study. 

Cytotoxicity studies with MTT assay, on the other hand, showed a moderate effect on the viability of the HT29-MTX cells after 24 h of exposure. The study was conducted on cells in an active growth phase that had not yet differentiated into mucus-producing cells, so mucus was not a factor. Although the study indicated that the formulations influenced cell viability, this was not a direct representation of in vivo conditions, as the concentrations represented and the time of exposure on the individual cells were higher than is likely to be the case in an in vivo situation. The viability after 2 h of exposure showed no statistical difference when compared with the untreated wells, indicating that short-term, direct exposure of SP-based formulations is likely to be non-toxic to cells. 

## 5. Conclusions

The CMC of Soluplus^^®^^ decreased with increasing temperatures from 25 °C to body temperature, as well as with the addition of furosemide, as determined by ITC. Furthermore, the thermodynamic properties of the furosemide–Soluplus^^®^^ micelles were influenced by the temperature, pH and composition of the test medium, showing increasing LCST from around 33 °C in PBS (pH 7.4) to 36 °C in Milli-Q water and 38 °C in 0.1 M HCl. The findings from the nano DSC studies were supported by the increasing polydispersity observed in the temperature scans by DLS. A strong concentration dependency was detected with respect to solubility, viscosity, micellar size and polydispersity in all three physiologically relevant aqueous media, providing insight into how Soluplus^^®^^ is likely to behave in oral drug delivery. The morphology of the micelles was confirmed with the use of TEM imaging. The drug release behaviour was found to be less affected by the drug loading than by the release media. The biocompatibility studies, in terms of the permeability of a paracellular marker after 24 h of exposure to Soluplus^^®^^ micelle systems of various concentrations, as well as MTT assay (2 and 24 h), both conducted in HT29-MTX cells, indicated low cytotoxicity. The inherent characteristics of the drug-loaded Soluplus^^®^^ micelles described in this study confirmed that Soluplus^^®^^ is well suited to be an oral nano carrier for poorly soluble drugs and can be employed as liquid and semi-solid systems in addition to the more studied solid systems.

## Figures and Tables

**Table 1:** Thermodynamic parameters from the ITC Analysis of the solubilisation of furosemide (FM) into Soluplus^^®^^ micelles; mean ± SD (*n* = 3).

Temperature (°C)	Sample Cell	CMC (mg/mL)	ΔH_mic_ (KJ·mol^-1^)	TΔS_mic_ (KJ·mol^−1^)	ΔG_mic_ (KJ·mol^−1^)
25 25	Milli-Q water 0.065 mM FM	0.8 0.5	26.3 ± 2.1 132.3 ± 4.6	55.7 168.7	−29.4 −30.6
37	Milli-Q water	0.5	12.6 ± 0.1	44.4	−31.8

**Table 2:** Calorimetric transition midpoint temperature (Tm) and enthalpy (ΔH) values obtained from nano DSC analysis of Soluplus^^®^^ micelles with and without solubilised furosemide for three media; mean ± SD (*n* = 3).

Test Medium	Furosemide (mg/mL)	Tm (°C)	ΔH (kJ·mol^−1^)
PBS pH 7.4	-	33.5 ± 0.3	176.0 ± 93.0
PBS pH 7.4	0.05	32.8 ± 0.6	158.5 ± 99.1
Milli-Q water	-	36.0 ± 0.1	145.6 ± 72.2
Milli-Q water	0.05	35.7 ± 0.2	139.5 ± 21.8
0.1 M HCl	-	38.2 ± 0.5	116.7 ± 23.0
0.1 M HCl	0.05	37.8 ± 0.6	174.6 ± 44.8

**Table 3:** The viscosity (mPa·s) of the furosemide-saturated Soluplus^^®^^ micelles over a range of concentrations in three different aqueous media at a temperature of 25 °C; mean ± SD (*n* = 3).

Conc. of Soluplus (% *w*/*w*)	Viscosity (mPa·s)		
	Milli-Q water	PBS pH 7.4	0.1 M HCl
0	0.96 ± 0.06	0.98 ± 0.01	0.97 ± 0.04
0.5	1.02 ± 0.01	1.03 ± 0.01	1.15 ± 0.14
2	1.17 ± 0.03	1.22 ± 0.03	1.23 ± 0.01
5	1.79 ± 0.02	1.76 ± 0.02	1.84 ± 0.04
7	2.21 ± 0.02	2.32 ± 0.04	2.49 ± 0.04
10	3.16 ± 0.01	3.87 ± 0.04	4.25 ± 0.06
15	4.28 ± 0.04	7.44 ± 0.54	10.33 ± 0.18
17	7.65 ± 0.03	11.26 ± 0.78	16.64 ± 0.47

## Supplementary Materials

The following is available at www.mdpi.com: Table S1. Amount of furosemide (FM) solubilised with increasing Soluplus^^®^^ concentrations determined after stirring for 96 h at room temperature (n = 3).
