# Supplementary material for: Micellisation Mechanism and Behaviour of Soluplus®–Furosemide Micelles: Preformulation Studies of an Oral Nanocarrier-Based System
Source: Pharmaceuticals (Basel). 2019 Jan 19;12(1):15. doi: 10.3390/ph12010015 (PMC6469188; doi:10.3390/ph12010015)
Supplement: Supplementary file 1 [file pharmaceuticals-12-00015-s001.pdf]

pharmaceuticals

Supplementary Material: Micellisation Mechanism and Behaviour of Soluplus®-Furosemide Micelles: Preformulation Studies of an Oral Nano-Carrier Based System

Julia F. Alopaeus, Ellen Hagesæther and Ingunn Tho

Table S1. Amount of furosemide (FM) solubilised with increasing Soluplus® concentrations determined after stirring for 96h at room temperature (n=3)

Conc. of Soluplus (% w/w)	Solubilised FM (mg/mL)		
	Milli-Q water	PBS pH 7.4	0.1 M HCl
0	0.01 ± 0.02	1.58 ± 0.42	0.003 ± 0.001
0.5	0.10 ± 0.15	1.71 ± 0.08	0.31 ± 0.05
2	0.81 ± 0.33	3.40 ± 0.82	1.21 ± 0.02
5	3.48 ± 0.17	6.47 ± 0.48	2.01 ± 0.10
7	x	7.61 ± 1.46	2.96 ± 0.54
10	5.75 ± 0.25	8.49 ± 1.18	3.26 ± 0.02
15	6.41 ± 0.31	9.76 ± 1.59	4.22 ± 0.44
17	8.55 ± 1.30	13.36 ± 1.36	5.88 ± 0.62
